# The Role of Diet and Gut Microbiome in Multiple Sclerosis

**DOI:** 10.7759/cureus.28975

**Published:** 2022-09-09

**Authors:** Maleesha Jayasinghe, Omesh Prathiraja, Abdul Mueez Alam Kayani, Rahul Jena, Dilushini Caldera, Minollie Suzanne Silva, Malay Singhal, Jimmy Pierre

**Affiliations:** 1 Medicine, Nanjing Medical University, Nanjing, CHN; 2 Medicine and Surgery, Nanjing Medical University, Nanjing, CHN; 3 Medicine and Surgery, Allama Iqbal Medical College, Lahore, PAK; 4 Medicine, Bharati Vidyapeeth Medical College/Bharati Hospital, Pune, IND; 5 Internal Medicine, Nanjing Medical University, Nanjing, CHN; 6 Internal Medicine, Mahatma Gandhi Memorial Medical College, Indore, IND

**Keywords:** intermittent fasting, obesity, mind diet, ketogenic diet, diet, fecal microbiota transplantation (fmt), microbiota, demyelinating dieases, gut-brain axis, multiple sclerosis

## Abstract

Multiple sclerosis (MS) is a chronic demyelinating condition of the central nervous system (CNS) characterized by immune-mediated damage to the myelin sheath of nerve cells. Genetic and environmental factors are believed to play a significant role. Unfortunately, the knowledge of therapeutic modalities in MS remains very limited, necessitating the need for novel therapeutic strategies. In the previous decade, there has been an influx of studies on the gut microbiome and its link to various neurological conditions, including MS. Various diets may have favorable effects on the gut microflora and may significantly alter the progression and outcomes of MS. Thus, identifying the merits of various diets and modulating them according to the specific nutritional requirements of MS patients can go a long way toward slowing the progression of the disease. Nutritional interventions and the use of the gut microbiome as diagnostic and therapeutic modalities open a host of new possibilities regarding the disease. In this review, we investigate the role of diet and the gut microbiome in the progression of MS. The functions of the gut-brain axis, antioxidants, vitamins, obesity, and various diets are also covered in this article.

## Introduction and background

Multiple sclerosis (MS) is a chronic immune-mediated inflammatory disease that results in demyelinating plaques involving the gray and white matter of the central nervous system (CNS) [1]. MS is believed to affect 2.8 million people worldwide, with an estimated prevalence rate of 35.9 per 100,000 people [2]. Although MS is known to affect both men and women, females are affected more than their male counterparts, with a ratio of 2.3 to 3.5:1 [3]. Although the exact etiology and pathogenesis of MS are not entirely understood, it is thought to result from focal immune cell infiltration in the white and gray matter of the CNS. The initiation and progression of MS are driven by the interaction between antigen-presenting cells and T-lymphocytes activated by helper T cells and adaptive immune responses. Various genetic and environmental factors can serve as risk factors for MS [4]. MS is further classified into the following four subtypes: relapsing-remitting MS (RRMS), primary progressive MS (PPMS), secondary progressive MS (SSPS), and clinically isolated syndrome (CIS) (Table 1).

**Table 1:** Classification of different subtypes of multiple sclerosis. MS: multiple sclerosis; CNS: central nervous system Original table prepared by Dr. Abdul Mueez Alam Kayani.

Subtype	Description
Relapsing-remitting MS: the most common type	Alternating periods of clearly defined new attacks or new neurological symptom relapses and partial or complete recovery remission
Clinically isolated syndrome	The first episode of neurological symptoms lasting at least 24 hours resulting from inflammation/demyelination in the CNS that does not meet the criteria for MS
Primary progressive MS	Worsening neurological function from the onset of symptoms without early relapses or remission
Secondary progressive MS	Characterized by a relapsing-remitting course, followed by a progressive worsening of neurological function with time

RRMS is the most common subtype and accounts for 87% of the total cases with unpredictable attacks followed by periods of remission [4]. The diagnosis of MS is based on the characteristic medical history, neurological examination, and diagnostic tests that include cerebrospinal fluid (CSF) examination, magnetic resonance imaging (MRI), and evoked potential testing with no specific serum biomarkers available at present [5]. Typically, MS presents as new neurological deficits that last more than 24 hours with anatomical localization in the absence of any infection [5]. There are no specific laboratory tests for MS. CSF examination demonstrates increased immunoglobulin concentration and two or more oligoclonal bands. MRI demonstrates characteristic lesions disseminated in time and space [6]. The most recent criteria for the diagnosis of MS is the 2017 McDonald Criteria depicted in Table 2 [5].

**Table 2:** 2017 McDonald criteria for the diagnosis of multiple sclerosis. MRI: magnetic resonance imaging Original table prepared by Dr. Abdul Mueez Alam Kayani.

Parameters	Clinical requirement
Dissemination in space	*More than or equal to one* T2-weighted lesion on MRI in *at least two out of four* locations: juxtacortical/intracortical, periventricular, infratentorial, or spinal cord
Dissemination in time	The simultaneous presence of symptomatic/asymptomatic gadolinium-enhancing and non-enhancing lesions or the presence of cerebrospinal fluid oligoclonal bands

Recent developments have led to the development of increasingly effective treatment options for RRMS and partially effective treatment options for PPMS and SSPS. With the development of much more effective treatment modalities, the mean time of 19 years for the progression of RRMS to SPMS has been lengthened [7]. Historically, MS has been treated with disease-modifying therapies (DMT) with an escalation approach, starting with modestly effective and progressing to highly effective DMT. The basic underlying principles of DMT in MS are to give patients the best chance of preventing future disability, including starting treatment shortly after initial symptoms and having a low threshold of switching therapies with a different mechanism of action in cases of breakthrough disease activity [8]. The currently approved different classes of DMT options for MS include interferons, glatiramer acetate, oral immunomodulators, cell migration modulators, and cell-depleting options [9].

Although treatment with DMT has shown some efficacy, these therapies are associated with their side effects. In some cases, they are more detrimental than the MS itself. The severe side effects of common DMT are summarized in Table 3 [10-12].

**Table 3:** Serious side effects of different disease-modifying therapies for multiple sclerosis. MS: multiple sclerosis Original table prepared by Dr. Abdul Mueez Alam Kayani.

Types of disease-modifying therapies	Serious side effects
Interferons: interferon β‐1a, pegylated interferon β‐1a, interferon β‐1b	MS aggravation, depression, suicide attempt, gait disturbance, dystonia, cerebral venous thrombosis
Glatiramer acetate	Nicolau’s syndrome (a cutaneous drug reaction characterized by ischemic necrosis of the skin and tissues), schizoaffective episode, breast cancer, cutaneous lymphoma, necrotizing cutaneous lesions
Oral immunomodulators: dimethyl fumarate, teriflunomide	Lymphopenia, leukopenia, progressive multifocal leukoencephalopathy, neutropenia, lymphopenia, hepatotoxicity, teratogenicity
Cell-migration modulators: natalizumab	Anaphylactic reaction, progressive multifocal leukoencephalopathy, rigidity, urinary tract infection
Cell-depleting therapies: alemtuzumab, cladribine	Immune thrombocytic purpura, herpes zoster, increased malignancy risk (malignant melanoma, pancreatic carcinoma, and ovarian carcinoma)

The risk of these severe side effects associated with DMT necessitates a closer examination of alternative therapies as a possible replacement for these traditional treatment options. There is an increased interest in the investigation of the possible role of nutritional interventions and gut microbiota playing a therapeutic role in the management of MS. Studies have demonstrated the role of a proinflammatory diet, for example, fatty acids (FA), polyphenols, and diets rich in carbohydrates and fats in the pathogenesis of MS [13,14]. The discovery that oxidative stress is one of the most important elements of the inflammatory process, leading to myelin breakdown and axonal damage, formed the basis for the use of antioxidants in MS. Therefore, it is critical to conduct additional research in this area to comprehend the protective effects of diets high in antioxidants in MS [15]. A high body mass index (BMI) before 20 years of age is strongly associated with an increased risk of MS [16].

The gut-brain axis (GBA) is found to play a crucial role in the effect of different types of diet in the pathogenesis of MS. It is a bidirectional communication consisting of neuronal signaling, neuroendocrine pathways, and modulation of the immune response. It may serve as a potential target in treating MS [1]. The human gastrointestinal tract (GIT) is a host to around 100 trillion microorganisms. The microbiota varies across ages. In addition, it varies between different individuals due to influences of BMI and external factors such as lifestyle, exercise frequency, ethnicity, and dietary and cultural habits [17]. In recent times, the role of commensal microbiota has gained immense interest in MS. Studies have shown that alterations of commensal microbiota act as an environmental risk factor for MS [18].

In light of the fact that the gut microbiota has emerged as a critical player in the GBA and a foundation for both health and disease, the use of therapeutic substances such as prebiotics, probiotics, fecal microbiota transplantation (FMT), and different types of diets to alter the gut microbiota opens up a potentially promising treatment option for diseases such as MS. Precision in microbiome identification, difficult strain selection for probiotics, and many prebiotic forms to identify the appropriate commensal have all raised the bar for future clinical application of microbiome-based therapy. Discoveries in gut microbiome research have made FMT possible as an alternative treatment for various clinical diseases, ranging from intestinal to extraintestinal microbiota-related illnesses. In addition to demonstrating safety and good tolerance, FMTs from healthy donors without a personal or family history of autoimmune illness have been demonstrated to lower intestinal permeability, change the cytokine balance to an anti-inflammatory state, and enhance the microbiota composition of RRMS patients [19]. More research is still needed to learn more about the clinical usage of FMT in treating MS.

The future uses of the microbiome in disease diagnosis, prognosis monitoring, prevention, and therapy have the potential to change current disease management and treatment techniques.

Concerning the function of diet in MS, research indicates that strict dietary patterns, such as the ketogenic diet (KD), are associated with a loss of patient follow-up, making it difficult to generalize these diets to the general population. Therefore, it is essential that we construct nutritional education programs that patients can easily adhere to over time. This can be accomplished through the use of a multidisciplinary team consisting of dietitians, nutritionists, neurologists, and other healthcare professionals to develop and implement nutrition education programs that adhere to best practice standards and utilize codesign to ensure participant needs are met. In addition, it is essential to conduct additional research on nutritional interventions for individuals with MS that take their needs and preferences into account and have the potential to improve their overall health and quality of life. This study will examine the therapeutic value of diet and microbiota in MS.

## Review

Gut-brain axis

The neuroanatomic, endocrine, humoral, metabolic, and immunologic bidirectional network between the GIT and CNS influences intestinal activity, immune cell activity, mood, cognition, and mental health [20-22]. The CNS can interact with the GIT via either sympathetic or parasympathetic components of the autonomic nervous system (ANS), the hypothalamic-pituitary-adrenal axis, and vagus nerves connecting to the visceral intestines linking the gut and the brain [20,21]. Recent research reveals that cross-talk along the GBA regulates inflammatory nociception, inflammatory responses, and immune homeostasis [23]. The pathways of the GBA have multiple interactions such as luminal gut microbiomes that can impact immunologic mast cell receptors and macrophages through the ANS. Various local neurotransmitters, such as gamma-aminobutyric acid (GABA), serotonin, melatonin, histamine, and acetylcholine, also act on the neurological pathway’s afferent sensory nerves [20]. The endocrine pathway of the GBA begins at the gut epithelium level. The gut epithelium consists of specialized chemosensory cells called enteroendocrine cells (EECs) that comprise less than 1% of the entire epithelial lining of the intestine and, when combined, make the gut a major endocrine organ in the body. Several unique EECs have been described based on their location and the hormones and neurotransmitters they secrete, such as the orexigenic hormone ghrelin, acid-modulating gastrin, and histamine. EECs elucidate the important neuroendocrine role in the GBA in regulating energy homeostasis and physiology [24]. Gut microbiota can be altered either directly by releasing biologically active molecules from the enteric nervous system or indirectly by modifying the microbial environment [25]. The “microbiota” refers to several resident microorganisms colonizing the gut that does not cause disease. The two most prominent bacteria in the gut microbiota are *Bacteroides *and *Firmicutes*. In healthy individuals, these bacteria, along with nutrient availability, influence the release of biologically active peptides from EECs, affecting the GBA [20,21]. In addition to playing several valuable functions in the human body, the gut microbiome plays an essential anti-inflammatory role by inhibiting histone deacetylase in regulatory T cells [26]. The human digestive system cannot digest dietary fibers, which allows microbes to ferment these nutrients into their absorbable form, especially certain carbohydrates, into short-chain FAs (SCFAs), which have anti-inflammatory and immunomodulatory effects [27]. These metabolites also affect the metabolic pathway of the GBA in multiple ways. SCFAs, bile acid metabolites, neuroactive substances (such as GABA, tryptophan precursors, serotonin), and other metabolic factors can affect the enterocytes directly and elicit neurocrine effects by interacting with nerve cells by enhancing the sympathetic branch of the ANS and can also have endocrine effects [20,21]. Most neurotransmitters cannot cross the blood-brain barrier (BBB), except for GABA (as GABA transporters are present); however, they may affect the brain indirectly by acting on the enteric nervous system [21]. Metabolites linked to the gut microbiota, such as SCFAs, can even cross the BBB and have been shown to regulate microglia homeostasis [20]. Inflammatory metabolites predominantly influence the immune pathway within the GIT produced by the gut microbiome, principally via the release of cytokines, such as interleukin (IL)-10, IL-4, and other cellular mediators, such as interferon-gamma, which can lead to inflammatory conditions [20].

Mental states of stress and depression have also been shown to impair the structure and function of the gut epithelial barrier. These states cause an increase in proinflammatory cytokines, such as IL-1 beta, IL-6, tumor necrosis factor-alpha, interferon-gamma, and C-reactive protein, resulting in increased intestinal epithelial permeability, allowing bacterial antigens and lipopolysaccharides to enter the circulation and elicit humoral responses within the body [20]. The gut microbiota helps maintain tight junction integrity between enterocytes. Increased permeability has been associated with gastrointestinal conditions, including irritable bowel syndrome and metabolic syndrome, as well as non-gastrointestinal conditions such as Alzheimer’s and asthma [20].

Modulating the GBA through various diets may play a significant role in the pathogenesis of MS by regulating the inflammatory response in the body. This review aims to explore the role of diet and its effects on the gut microbiome in the context of MS.

Types of diet

Existing evidence suggests that nutrition and various diets play a significant role in the pathogenesis of MS. Changes in diet have been shown to affect the activity and progression of MS. Below, we have discussed how different types of diets trigger MS-related inflammation and demyelination (Figure 2).

**Figure 1:**
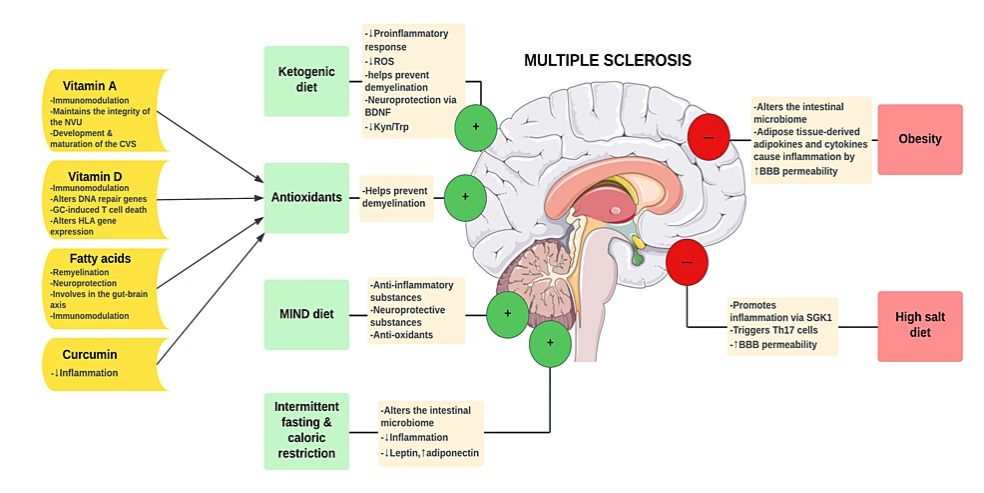
The role of diet in multiple sclerosis. GC: glucocorticoid; NVU: neurovascular unit; ROS: reactive oxygen species; CVS: cerebrovascular system; BDNF: brain-derived neurotrophic factor; Kyn/Trp: kynurenine/tryptophan; MIND: mediterranean-DASH intervention for neurodegenerative delay; BBB: blood-brain barrier; SGK-1: serum- and glucocorticoid-regulated kinase 1; Th17: T helper 17 cells Original figure prepared by Maleesha Jayasinghe.

Dietary Antioxidants

Antioxidant factors in the diet may regulate the activation of immune-inflammatory cells, decreasing inflammation. They may also mitigate oxidative stress, preventing chronic demyelination and axonal damage. Antioxidant factors, such as vitamin D, vitamin A, curcumin, and FA, appear to regulate oxidative stress, which was found to play a role in MS [13].

Vitamin D

Vitamin D is a lipid-soluble hormone that can be produced photochemically in the skin and consumed in food. The two most prevalent forms of vitamin D are D3 (cholecalciferol) and D2 (ergocalciferol). D3 is derived from sun exposure and the consumption of fish, milk, and plants, while D2 is derived from plant consumption. Both forms are converted to 25-hydroxyvitamin D and stored in the liver prior to being activated in the kidney to 1,25-hydroxyvitamin D. Multiple clinical studies have found a correlation between vitamin D levels and the progression of MS, which may be attributable to the essential immunomodulatory function of vitamin D. Because most immune cells express the vitamin D receptor and 1-hydroxylase, vitamin D influences the phagocytic activity of macrophages and natural killer (NK) cells. In addition, it increases the activity of phagocytes involved in microbicidal defense. Vitamin D inhibits the differentiation of antigen-presenting dendritic cells and B lymphocytes, as well as type 1 T helper (Th1) and Th17 cell proliferation. Vitamin D deficiency is believed to contribute to the development of cytotoxic T lymphocytes (CTLs), dysfunctional T helper cells, NK cells, and B cells in the CNS, which causes inflammation that damages neurons and oligodendrocytes in MS [28].

A recent study conducted in 2021 revealed that vitamin D supplementation might play a role in MS patients via DNA repair genes, making it a valuable therapeutic target for MS patients. This study demonstrated that vitamin D supplementation for two months in vitamin D-deficient MS patients led to a significant increase in the expression of MutT homolog 1, 8-oxoguanine glycosylase, and nuclear factor erythroid 2-related factor 2 [29].

Hoepner et al. demonstrated that 1,25-dihydroxyvitamin D3 increased glucocorticoid receptor (GR) protein levels in vitro, which resulted in an increase in glucocorticoid-induced T cell death. Based on the T cell GR expression, the combination of 1,25-dihydroxyvitamin D3 and glucocorticoids improved the therapeutic outcome of experimental autoimmune encephalomyelitis (EAE) more than the individual monotherapies. EAE is an animal type of MS that has been essential in developing various treatments for MS. The mammalian target of the rapamycin (mTOR) pathway was found to be responsible for the synergistic effects of 1,25-dihydroxyvitamin D3/glucocorticoid combination therapy on apoptosis induction. Low concentrations of 1,25-dihydroxyvitamin D3 were associated with a reduction in the expression of mTORc1, inhibiting tuberous sclerosis 1 in CD8+ T cells. In vitro and in vivo, 1,25-dihydroxyvitamin D3 promotes glucocorticoid-mediated effects in T cells by inhibiting mTORc1. These findings could aid in enhancing the anti-inflammatory effects of glucocorticoid therapy [30].

Until now, CHOLINE is the largest two-year randomized controlled trial conducted on MS patients to demonstrate the effects of cholecalciferol on patients with RRMS. It was constructed with a careful selection of patients to avoid the limitations of previous research. The study employed three criteria for selection. Annual relapse rate (ARR) was the primary endpoint, so the first objective was the recruitment of clinically active RRMS patients. Second, cholecalciferol was administered to patients already receiving interferon beta-1a treatment due to its synergistic or additive effects on blood monocytes and MS activity parameters. Third, a low 25-hydroxy vitamin D blood level was required for inclusion. Consistent with previous research, the present study demonstrated that cholecalciferol at 100,000 IU every two weeks was well tolerated. The study’s primary endpoint, a reduction in the ARR, was not demonstrated in the population intended to receive treatment. In contrast, treatment with cholecalciferol was associated with a lower ARR, a reduction in the number of new T1 lesions, a decrease in the volume of hypointense T1-weighted MRI lesions, and a significantly slower progression of expanded disability status scale (EDSS) in the completers’ population. Adjusted baseline, the number of relapses decreased significantly with increased mean 25-hydroxy vitamin D concentrations, and a benefit on EDSS progression was also observed [31].

Human leukocyte antigen (HLA) alleles such as HLA-DRB1*1501 significantly increase the risk of developing MS. Recent studies have identified vitamin D response elements in the promoter region of the HLA-DRB1 gene. The activation of the vitamin D receptor by 1,25(OH)2D may alter the expression of this gene, thereby reinforcing the association between vitamin D and MS [28].

Vitamin A

Retinoic acid, a derivative of vitamin A, maintains transcription at the level of the nucleus. It plays a vital role in the development and maturation of the cerebral vascular system and the maintenance of neurovascular unit (NVU) integrity. Neurons, glial cells, and vascular cells make up the NVU. The NVU ensures proper BBB function and homeostasis of the CNS. Consequently, retinoic acid maintains the structural integrity of the neurovascular unit, thereby preventing the development of neurological disorders. Multiple neurodegenerative diseases, such as Parkinson’s disease, Alzheimer’s disease, and MS, can develop due to the deletion of retinoic acid. Retinoic acid promotes neuronal regulation and differentiation, modulates the inflammatory response of astrocytes and microglia, and controls endothelial-pericyte interactions. Vitamin A modulates immune function through its pleiotropic effects on innate and adaptive immune systems, making it a promising candidate for application to MS patients. Retinoic acid supplementation in mice deficient in vitamin A has been shown to stimulate the induction of T regulatory cells. Retinoic acid inhibits the expression of retinoid-related orphan nuclear receptor t (RORt), an orphan receptor related to retinoic acid receptors. RORt is a crucial transcription factor required for the differentiation of Th17 and the production of cytokines of the IL-17 family. Several autoimmune pathologies have been associated with dysregulated Th17 immune responses. Vitamin A and its derivatives reduce neuroinflammation by inhibiting leukocyte proliferation and decreasing IL-2 production. In EAE, it also inhibits leukocyte proliferation and induces a regulatory Th2 immune response. Reports have shown that MS patients have lower levels of retinol and beta carotene (vitamin A status indicators) than healthy controls. In RRMS patients, serum retinol has also been found to be inversely associated with MRI lesions. The combination of all-trans retinoic acid and interferon-beta has an additive effect on restoring suppressor T cell function in the peripheral blood mononuclear cells of MS patients [32].

Bitarafan et al. conducted a placebo-controlled, randomized clinical trial with 101 patients with RRMS to determine the effect of vitamin A on disease progression in MS patients. The treatment group was given 25,000 IU/day of retinyl palmitate for six months, followed by 10,000 IU/day for another six months. During the intervention, the relapse rate was recorded along with the EDSS and MS functional composite (MSFC) results. In addition, patients underwent both baseline and follow-up brain MRIs. In the treatment group, the MSFC score improved significantly, whereas the EDSS score remained unchanged. No changes in gray matter volume were observed in the patients, but this could be due to the limitations of the MRI protocol. Vitamin A did not significantly alter the relapse rate in MS patients [33].

In addition, studies have shown that the co-administration of vitamins A and C decreases the levels of proinflammatory cytokines and inducible nitric oxide synthase and increases gene expression of IL-10, nuclear factor erythroid 2-related factor 2, heme oxygenase-1, and myelin basic protein. The combination of vitamins A and C increases the total antioxidant capacity and reduces the levels of oxidative stress markers. Navidhamidi et al. demonstrated that the co-administration of vitamins A and C has anti-apoptotic and neuroprotective effects in EAE by brain-derived neurotrophic factor (BDNF), decreasing caspase-3 and increasing the number of cells expressing BDNF and neuronal nuclear protein. This study suggests combining vitamins A and C may be an effective strategy for developing alternative medicine to enhance myelin repair in demyelinating diseases [34]. Given the evidence supporting the immunomodulatory role of vitamin A in preclinical models and the growing literature suggesting a possible benefit of vitamin A supplementation in MS patients, it is essential to conduct further clinical studies to understand the role of vitamin A in MS.

Fatty Acids

FAs can function as biomarkers of disease activity and therapeutic efficacy in MS. Because FAs are significant components of oligodendrocyte membranes, systemic and CNS resident FA levels fluctuate significantly during demyelination and remyelination. Multiple FA concentrations can reflect disease activity, including intestinal barrier permeability, the regulatory T cells (Treg)-Th1 axis balance, and the EDSS score. The consumption of FAs or the metabolism of FAs contributes to susceptibility to MS. Before disease onset, MS patients acquire a unique FA serological profile. According to data from the microbiome, MS patients have a decrease in SCFA-producing bacteria. SCFAs and medium-chain FA mediate communication between the gut microbiota and the immune system by exerting protective or damaging effects. Consequently, FAs can be essential therapeutic targets for restoring GBA disruption. MS is associated with several FA metabolism-related enzyme single nucleotide polymorphisms (SNPs) and polyunsaturated FA (PUFA) intake patterns. FAs and related bio-mediator levels are altered in MS patients; consequently, FA supplementation reduces the incidence, ARR, clinical score, CNS pathology, and quality of life (QoL) of MS patients, making FAs a potential therapeutic target for MS. Biological constitutions of FAs are constituents of MS metabolic memory that would influence the immune system. MS patients with significantly decreased adipose-resident oleic acid have a proinflammatory transcriptional profile of Treg cells, which can be reversed by supplementation with oleic acid [35].

Omega-3 PUFAs are antioxidants linked to ameliorating neurodegeneration in MS. PUFAs found in fish, nuts, and seeds appear to be associated with protection against demyelination. In animal models, they were found to reduce inflammation, maintain immunomodulation, and promote neuroprotection and remyelination. PUFA-rich diets have been associated with a low incidence of MS, and meta-analyses have shown a reduction in the frequency of relapses but no effect on the progression of MS [13]. Matrix metallopeptidase-9 (MMP-9) is an example of a specific marker associated with inflammation and neurodegeneration in patients with MS. PUFAs were connected to an improvement in the levels of MMP-9 in a study by Esposito et al. Among PUFAs, alpha-linolenic acid (ALA) is linked to a reduced incidence of MS [36]. It can contribute to the immune pathway by reducing inflammatory markers. Eicosapentaenoic acids (EPAs) and docosahexaenoic acids (DHAs) may also reduce MMP-9 levels in MS patients. Riccio et al. reported that supplementation of fish oil containing omega-3 FA has a beneficial effect on the inhibition of expression of MMP-9 levels in MS patients. The supplementation of omega-3 FA reduces proinflammatory cytokines and free radicals, thereby improving the QoL of MS patients by reducing relapse rates [13].

PUFAs have been found to not only improve the clinical outcomes of MS patients but also cause an improvement in the functional capacities and gait parameters of RRMS patients. A recent randomized controlled trial conducted on 51 patients with RRMS demonstrated that a unique dietary formula (Neuroaspis®PLP10), a combination of specific bioactive molecules, the omega-3 PUFAs DHA and EPA, the omega-6 PUFAs linoleic acid and gamma-linolenic acid, and several vitamins such as vitamin E and γ-tocopherol, can act protectively against functional deterioration of patients with RRMS [37].

Curcumin

Curcumin, a compound derived from the plant *Curcuma longa*, has been proposed as an inhibitor of proinflammatory cytokines. In animal models of MS, curcumin was found to decrease clinical severity and CNS infiltration of inflammatory cells in mice. Curcumin has anti-inflammatory and antioxidant properties. Its antioxidant effects have been evaluated in various neurodegenerative diseases, including Alzheimer’s, Parkinson’s, and MS [13].

Obesity

Obesity is characterized as a BMI of 30 kg/m^2^ or above. It is associated with multiple health consequences such as type 2 diabetes mellitus, reduced levels of vitamin D, lipid profile abnormalities, hypertension, and a low-grade inflammatory state in the CNS which can lead to the development of MS. This could be attributed to the increased BBB permeability by the actions of adipose tissue-derived cytokines or adipokines. By crossing the BBB, proinflammatory adipokines such as leptin, resistin, and visfatin activate CNS-resident immune cells, promote inflammatory responses, and cause demyelinating lesions in the white matter of the brain and spinal cord. Consequently, detailed knowledge of the functions of adipokines in the generation of obesity-related chronic inflammation and subsequent events leading to BBB failure is crucial [38].

**Figure 2:**
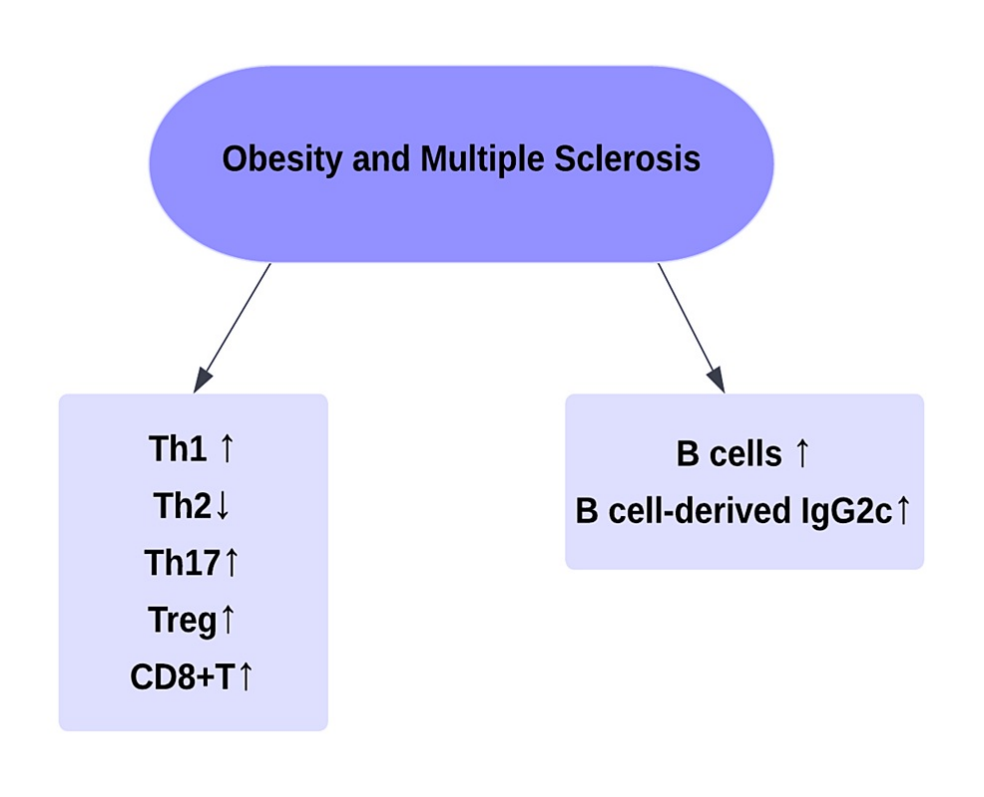
Changes in immunological markers in obesity and multiple sclerosis. Th1: T helper cell type 1; Th2: T helper cell type 2; Th17: T helper 17 cells; Treg: regulatory T cells Original figure prepared by author Maleesha Jayasinghe.

A study conducted by Schreiner et al. proposed three theories regarding the association between obesity and MS: the inflammatory theory, the hormonal theory, and the intestinal gut microbiome theory [39]. The inflammatory theory refers to the changes in immunological markers observed in obese patients that are similar to the changes encountered in the pathogenesis of MS (Figure 2).

The function of B cells in the pathogenesis of MS has recently become a focal point of research, with theories positing that both antibody-dependent and independent factors play a role in the initiation of demyelination. Demyelination may be caused by the production of specific autoantibodies against CNS structures, which activate T cells, or the formation of ectopic lymphoid structures, which generate cytokines and neurotoxic chemicals. The hormonal theory primarily focuses on how adipokines such as leptin, adiponectin, and resistin function as biomarkers in obese MS patients. The relationship between adipokine production, obesity, and MS is intriguing yet little understood. Studies suggest that the number of adipokines in the blood and cerebrospinal fluid may be associated with the different forms of MS, its progression, and the degree of disability. Thus, adipokines may play a role as potential biomarkers for early disease detection and monitoring disease activity [39]. Leptin, encoded by the LEP gene, is primarily produced by adipocytes, so BMI determines its levels. It exerts physiological activity via a member of the class I cytokine receptor family (leptin receptors). This receptor regulates body weight by reducing food intake and increasing energy expenditure by inducing anorexigenic hormones and suppressing orexigenic neuropeptides in the hypothalamus. However, leptin also regulates autoimmune and inflammatory processes, suggesting a link between metabolism and immune response. According to a study conducted by Marrodan et al., leptin plays a dual role in MS patients. On the one hand, it promotes inflammation by increasing CD4+CD25MBP and myelin oligodendrocyte glycoprotein‐peptide‐specific T‐cell proliferation, promoting proinflammatory cytokine secretion, and inhibiting apoptosis in myelin-reactive T cells. On the other hand, leptin inhibits CD4+ CD25+ Treg cell proliferation, causing these cells to become hyporesponsive. Consequently, leptin-deficient (obese mouse) mice were resistant to the onset of experimental autoimmune encephalomyelitis (EAE), which the administration of exogenous leptin reversed. Notably, decreased food intake and body weight precede the appearance of neurological symptoms following EAE induction. These changes are associated with a marked increase in circulating leptin levels and leptin secretion by T cells in active EAE brain lesions. Similarly, research has shown that serum and CSF leptin levels are elevated in RRMS patients, both in correlation with interferon secretion in the CSF and a reduction in circulating Treg cells [40]. Duscha et al. administered exogenous propionic acid as an add-on therapy to a study group. They monitored immunological and disease activity to demonstrate the role of propionic acid, a FA found in low concentrations in the stool of MS patients, in the modulation of disease activity. A reduction in relapse rate was associated with a decrease in Th1 activity and an increase in Treg function [39]. Further clinical studies are needed to gain a better understanding of the role of the gut microbiome in MS.

Ketogenic Diet

The KD is a high-fat, low-carbohydrate diet that stimulates the synthesis of ketone bodies as a source of energy. By lowering Th1 and Th17 responses, reducing reactive oxygen species (ROS) generation, and increasing Treg cell responsiveness, KDs inhibit EAE. Several pilot studies on RRMS have demonstrated a considerable improvement in QoL, fatigue, and depression over three to six months with a KD [32].

A study conducted on mice by Kim et al. revealed that KD is crucial for neuroprotection. According to the study, the EAE mouse model fed a KD with a fat-to-carbohydrate-to-protein ratio of 6.30:1 exhibited improved spatial learning and memory, enhanced motor skills, and reduced lesion size. Additionally, these improvements were linked to a decrease in ROS production and a reduction in the proinflammatory responses in brain tissue (cytokines, chemokines, macrophages, and microglia). A more tolerable KD with a fat-to-carbohydrate-plus-protein ratio of 3:1 showed neuroprotection in the cuprizone-induced demyelination mouse model of MS. The KD ameliorated motor and behavioral deficits, increased the number of mature oligodendrocytes, and decreased hippocampal demyelination in animals. In addition, the increased expression of myelin basic protein and other myelin-associated markers suggests that the KD is protective against demyelination [41]. In addition to its neuroprotective effects, KD was observed to improve body composition, fatigue, depression, QoL, and adipose-related inflammation in 65 persons with relapsing MS who participated in a prospective six-month KD intervention with the purpose of treating [42].

With the identification of the role of impaired myelination in MS, the significance of neurotrophic growth factors in myelin repair has been highlighted. BDNF is the gold standard among them in this context. Neurodevelopment, neuronal function, and survival in the adult brain, as well as synaptic plasticity in the hippocampus, depend on BDNF. Several effects of BDNF have been demonstrated to contribute to neuroprotection. It may affect the distribution pattern of myelin structural proteins that contribute to the integrity of an intact myelin sheath. This myelin-protective effect helps ensure mechanisms of myelin repair and, ultimately, the degree of remyelination by endogenous pathways induced by BDNF. It has been discovered that it induces oligodendrocyte precursor proliferation, migration, and differentiation in myelin damage foci. BDNF also serves as a neuronal survival factor by promoting the remyelination of damaged axons. BDNF is also involved in glucidic homeostasis and energy balance, as an inverse correlation was found between blood glucose levels and BDNF release. Interestingly, KD was linked explicitly to the modification of plasma BDNF levels. The dietary protocol’s ability to produce beta-hydroxybutyrate (BHB) allows it to pass through the BBB, increasing mitochondrial respiration and, in turn, activating NF-KB, which, in turn, activates histone acetyltransferase p300/EP300 and, ultimately, BDNF synthesis. The effect of the kynurenine/tryptophan (Kyn/Trp) ratio on inflammatory states and neuronal excitability was evaluated, revealing that lowering the Kyn/Trp ratio in favor of Trp via the consumption of Trp-rich foods improves skeletal muscle mass and reduces endogenous inflammation in MS patients. The manipulation of Trp-Kyn metabolism by lifestyle factors (diet, branched-chain amino acids, aerobic activity) could tip the scales in favor of Trp and its neuroprotective intermediates, thereby aiding in the treatment of MS with low-grade chronic inflammation. BHB created during the KD regimen results in a drop in Kyn levels, an increase in kynurenic acid (KA), and the KA/KYN ratio, which maintains the neuroprotective benefits of KD by blocking the enzyme kynurenine 3-monooxygenase, which is ultimately responsible for producing KA [43].

High-Salt Diet

Recent attention has been given to the effect of dietary salt intake on MS, given the excessive salt consumption characteristic of the western diet and the prevalence of MS in the western world. Hypertonic saline was found to activate immune cells and stimulate the production of proinflammatory cytokines [32]. A high-salt diet has been demonstrated to trigger the development of Th17 and promote inflammation via serum glucocorticoid kinase 1 (SGK1). SGK1 is a serine-threonine kinase that plays a significant role in regulating IL-23R expression and stabilizing the Th17 cell. It governs Na+ transport and salt homeostasis in other cells [44].

A high-salt diet aggravates actively induced EAE and shows enhanced CNS infiltration and peripherally induced pathogenic Th17 cells. Another study highlighted the role of the gut microbiota in the exacerbation of EAE. Supplementation with *Lactobacillus murinus* or *Lactobacillus reuteri* blunted salt-induced pathogenic Th17 cells and ameliorated BBB permeability. This effect might be related to endothelial cells’ decreased tight junction (TJ) proteins or due to the upregulation of serum corticosterone and tightening BBB [45].

However, data from humans suggested that the high salt effect on MS is controversial; therefore, further clinical studies are required to gain a better understanding of the effects of a high-salt diet on the pathogenesis of MS. One study reported that high salt intake was associated with increased disease activity in MS patients while four other studies found no association between HSD and MS progression. However, these clinical studies utilized spot urine collections and food questionnaires to measure dietary salt intake, which may have led to invalid results; therefore, more accurate measurement methods are required to measure salt intake in future studies [45].

Mediterranean-DASH Intervention for Neurodegenerative Delay Diet

The Mediterranean-DASH Intervention for Neurodegenerative Delay (MIND) diet encourages natural plant-based meals and restricts the consumption of animal-derived or high-saturated fat foods. It promotes the consumption of berries and green leafy vegetables, which are high in anti-inflammatory, antioxidant, and neuroprotective substances. Morris et al. introduced the MIND diet, a combination of the Mediterranean and DASH diets. Due to its neuroprotective properties, the MIND diet was associated with a reduction in the development of MS. According to research, the polyphenols found in vegetables and beans may inhibit the production of proinflammatory factors. A high intake of the recommended products of this diet, such as vegetables, fruits, and fish, combined with a low intake of less healthy foods among participants in this study was insufficient to ensure a positive influence on their metabolic health due to the effects of low-quality foods with insufficient amounts of antioxidants and anti-inflammatory substances [46].

Greater adherence to the MIND diet has been associated with a reduced risk of cognitive decline. However, additional research is required to acquire a comprehensive knowledge of the relationship between the MIND diet and MS. This retrospective analysis demonstrates that greater adherence to the MIND diet may protect against MS. Individuals in the third tertile of MIND scores were estimated to be 90% less likely to have MS than those in the first tertile. Those in the middle tertile, on the other hand, had an estimated 84% lower risk. Other factors, including diet and BMI, did not affect the study results. However, the consumption of harmful components of the MIND diet, such as butter and stick margarine, was related to decreased odds, although this result may be attributable to the misclassification of soft and hard margarine as a category. In addition, a large intake of cheese, pastries, sweets, and fast food increases the likelihood of developing MS. As a nutritional component, poultry was also related to increased probabilities. Therefore, it appears that adhering to the MIND diet may be an effective strategy for MS prevention [47]. However, additional randomized clinical trials are required to advocate the MIND diet as a preventative measure for MS.

Intermittent Fasting and Caloric Restriction

Intermittent fasting (IF) refers to periods with restricted or no caloric intake, that is, voluntary abstinence from food and drink (periods of fasting). During the fasting period, caloric intake typically ranges from 0-25% of the standard caloric requirements. IF may be practiced with unrestricted consumption or in conjunction with other dietary interventions. Since the 1960s, fasting has been regarded as an effective treatment for obesity and comorbidities. It is essential to note that IF has been practiced for thousands of years for health and religious reasons, such as during the month of Ramadan. IF is compatible with daily life and may be adopted as a lifelong eating pattern. There is evidence that IF regulates the levels of various proteins involved in lipid metabolism. It increases the lipolysis of triglyceride in chylomicrons and the production of high-density lipoprotein-cholesterol, resulting in a significant decrease in total plasma triglycerides. Early time-restricted feeding is helpful for 24-hour glycemic control. Furthermore, it increases hydroxybutyrate, lowers oxidative stress, maintains lean mass, and lessens the sensation of hunger. The composition of the intestinal microbiome is altered by IF, which enriches the Bacteroidaceae, Lactobacillaceae, and Prevotellaceae families. The abundance of lactobacilli caused by IF has beneficial effects, such as reducing inflammatory immune responses and aiding in treating MS patients [48].

MS patients on an intermittent caloric restriction (CR) diet exhibited a decrease in memory T cells and lipid markers and an increase in naive T cells. Fasting causes a shift toward Treg cells and away from effector T cells, in addition to a rise in the proportion of naive T cells [37]. Choi et al. conducted a study in which 17 MS patients recovering from relapse were randomly assigned to alternate-day fasting or their usual diets for 15 days. In contrast to the study conducted by Fitzgerald et al., intermittent CR was associated with a decrease in the number of naive CD4+ and CD8+ T cells and an increase in the suppression of the function of Treg cells (the number of Treg cells remained unchanged) [49]. However, it is crucial to note that all participants in this study were administered glucocorticoids, which may have altered the lymphocyte composition. Not only could the shorter study duration (15 days) have contributed to the disparate results compared to the study conducted by Fitzgerald et al. but the shorter duration may have also played a role. Despite several prior studies suggesting a correlation between fasting and a decrease in leptin levels and an increase in adiponectin, neither leptin nor adiponectin levels changed [50]. This result could be attributed to the study’s relatively small sample size. Beyond the 5:2 diet evaluated here, different fasting protocols (alternate-day fasting, time-restricted feeding) may also have different effects on adipokines and other immune cell parameters. Prior mechanistic investigations also showed a relationship between leptin and T cell activation and proinflammatory polarization, indicating that leptin may mediate the effects of fasting on the relative distribution and function of T cells. Independent of leptin, the results of this study suggest that intermittent CR may affect T cell subset changes. As alterations in specific lipid metabolites can shift the distribution of T cells away from effector T cells, it is possible that some of the observed changes in lipid metabolites mediate some of the observed associations between intermittent CR and changes in the relative composition of T cells. Low levels of glycerophospholipids and lysoplasmalogens may impact the functionality of T cell subpopulations, such as NK cells, that are activated by these endogenous lipids. Considering that these T cell populations can produce other proinflammatory mediators that can influence T cell function, this may explain the immunological changes observed in our study [50].

Table 1 is a compilation of clinical trials discussing the effect of various diets on the progression of MS.

**Table 4:** Compilation of clinical trials discussing the effect of various diets on the progression of MS. MS: multiple sclerosis; RRMS: relapsing-remitting multiple sclerosis; ARR: annualized relapse rate; EDSS: expanded disability status scale; MSFC: Multiple Sclerosis Functional Composite; GDI: gait deviation index; STS: sit-to-stand-to-sit; 6MWT: six-minute walk test; TUG: Timed Up and Go; EMG: electromyography; QoL: quality of life; MIND: Mediterranean-DASH Intervention for Neurodegenerative Delay; CR: caloric restriction

Author	Type of study	Sample size	Type of diet	Result	Study weaknesses
Amirinejad et al. [29]	Clinical trial	31	Vitamin D	MYH, OGG1, MTH1, and NRF2 gene expression in MS patients treated with vitamin D for two months was significantly altered	Mononuclear cells were not sorted. Limited samples from RRMS patients due to the exclusion of some volunteer patients in the second phase of the study
Camu et al. [31]	Double-blind, placebo-controlled, parallel-group study	181	Vitamin D	The primary endpoint of the study was not met. There was a reduction in ARR, fewer new hypointense T1-weighted lesions, a lower volume of hypointense T1-weighted lesions, and a slower progression of EDSS in patients who completed the two-year follow-up	Statistical power - there was difficulty in achieving the inclusion target patient number. The sample size was determined based on a relapse rate of one per year, which was an overestimate given that interferon beta-1a was anticipated to minimize the relapse rate. Dropouts were underestimated
Bitarafan et al. [33]	One1-year placebo-controlled, randomized clinical trial	101	Vitamin A	Total MSFC score improved significantly in RRMS patients, although recurrence rate, EDSS, and brain active lesions remained the same	No change in brain active lesions was seen, which may have been due to limitations in the MRI methodology. Short follow-up period
Aristotelous et al. [37]	Randomized controlled trial	51	Fatty acids	The single support time and the step and stride times showed significant improvement. At 24 months, the GDI had increased by around 4%. There was a performance improvement in the STS-60 test and a trend toward improvement in the 6MWT and TUG tests	This study assessed the subjects’ natural, self-selected walking speed. A higher enforced walking speed is more likely to expose group differences. The isokinetic dynamometer only tested the maximum strength of knee extensors and flexors; however, to complete the STS movement, a number of muscle groups must be engaged during the required motions. During the functional testing, an EMG was not used to evaluate the activity of the trunk, ankle, knee, and hip muscles. Small sample size
Brenton et al. [42]	Prospective, intention-to-treat cohort	65	Ketogenic diet	There were considerable decreases in fat mass and a decrease of approximately 50% in self-reported levels of fatigue and depression. Diet improved MS QoL physical health and mental health composite scores. The scores on the EDSS, the 6-minute walk, and the Nine-Hole Peg Test improved significantly. On the KD, serum leptin was lower and adiponectin was greater	Small sample size
Noormohammadi et al. [47]	Case-control study	225	MIND diet	The RRMS and control groups differed significantly in median age, BMI, and total intake of calories, carbs, animal-based protein, and fiber. The MIND diet was connected with reduced odds of MS. In the last tertile of consumption for green leafy vegetables, other vegetables, butter, stick margarine, and beans, the risks of MS were considerably lower. While it was considerably higher in the last tertile of cheese, chicken, pastries and sweets, and fried/fast foods	Participants were not age-matched and were selected from the hospital. Patients with MS from the hospital are not generalizable to the community. The study lacked data on education levels, socioeconomic status, physical activity, and previous infection or vaccination history. It would also be better to consider information about other factors, such as the serum level of vitamin D
Fitzgerald et al. [50]	Randomized controlled study	36	Intermittent caloric restriction	Significant reduction in effector memory and proportional increases in the fraction of naive groups. In intermittent CR, more significant within-person variations in lysophospholipid and lysoplasmalogen metabolites were related to a greater reduction in memory T cell subsets and a greater increase in naive T cell subsets	A short sample size precludes assessing effect change by immunotherapy type. The short duration of the study. Participants in this trial were required to be on a first-line MS therapy or receive no treatment; the results may not be applicable to MS patients receiving more potent immunotherapies. The relatively small number of men and non-white participants may limit the generalizability of the findings. All analyses were conducted on frozen samples, which could have impacted T cell, metabolite, and adipokine analysis. The feeding research was also conducted remotely, with self-reported adherence data

Limitations

Our paper depends on a survey of open-access research journals produced during the past decade; therefore, we may have excluded significant material from paid full-text journals and research articles published before 2015. Moreover, as the focus of our analysis is limited to English-language studies, we may have missed papers published in other languages.

## Conclusions

MS is a chronic inflammatory disease characterized by demyelinating plaques in the gray and white matter of the CNS. Genetic and environmental factors are crucial to the pathogenesis of MS. It has been discovered that the GBA regulates the inflammatory response and immune homeostasis, thereby contributing to the development of MS. Recent research indicates that dietary changes can alter the gut microbiome, triggering inflammation and demyelination in MS. In RRMS, the KD has shown an improvement in QOL, fatigue, and depression. In contrast, the MIND diet has demonstrated a decrease in MS incidence and cognitive decline. These studies highlight the importance of considering the role of diet and gut microbiota in the therapeutic management of patients with MS.
